# *Blastocystis* One Health Approach in a Rural Community of Northern Thailand: Prevalence, Subtypes and Novel Transmission Routes

**DOI:** 10.3389/fmicb.2021.746340

**Published:** 2021-12-09

**Authors:** Vasana Jinatham, Sadiya Maxamhud, Siam Popluechai, Anastasios D. Tsaousis, Eleni Gentekaki

**Affiliations:** ^1^School of Science, Mae Fah Luang University, Chiang Rai, Thailand; ^2^Laboratory of Molecular and Evolutionary Parasitology, RAPID Group, School of Biosciences, University of Kent, Canterbury, United Kingdom; ^3^Gut Microbiome Research Group, Mae Fah Luang University, Chiang Rai, Thailand

**Keywords:** asymptomatic hosts, *Blastocystis*, environmental transmission, One Health, rural community, Thailand

## Abstract

*Blastocystis* is the most commonly found eukaryote in the gut of humans and other animals. This protist is extremely heterogeneous genetically and is classified into 28 subtypes (STs) based on the small subunit ribosomal RNA (*SSU* rRNA) gene. Numerous studies exist on prevalence of the organism, which usually focus on either humans or animals or the environment, while only a handful investigates all three sources simultaneously. Consequently, understanding of *Blastocystis* transmission dynamics remains inadequate. Our aim was to explore *Blastocystis* under the One Health perspective using a rural community in northern Thailand as our study area. We surveyed human, other animal and environmental samples using both morphological and molecular approaches. Prevalence rates of *Blastocystis* were 73% in human hosts (*n* = 45), 100% in non-human hosts (*n* = 44) and 91% in environmental samples (*n* = 35). Overall, ten subtypes were identified (ST1, ST2, ST3, ST4 ST5, ST6, ST7, ST10, ST23, and ST26), eight of which were detected in humans (ST1, ST2, ST3, ST4, ST5, ST7, ST10, and ST23), three in other animals (ST6, ST7, and ST23), while seven (ST1, ST3, ST6, ST7, ST10, ST23, and ST26) were found in the environment. In our investigation of transmission dynamics, we assessed various groupings both at the household and community level. Given the overall high prevalence rate, transmission amongst humans and between animals and humans are not as frequent as expected with only two subtypes being shared. This raises questions on the role of the environment on transmission of *Blastocystis*. Water and soil comprise the main reservoirs of the various subtypes in this community. Five subtypes are shared between humans and the environment, while three overlap between the latter and animal hosts. We propose soil as a novel route of transmission, which should be considered in future investigations. This study provides a thorough One Health perspective on *Blastocystis*. Using this type of approach advances our understanding on occurrence, diversity, ecology and transmission dynamics of this poorly understood, yet frequent gut resident.

## Introduction

*Blastocystis* is the most ubiquitous protist inhabiting the gastrointestinal tract of human and other animal hosts (Roberts et al., 2013; Beghini et al., 2017; Stensvold and van der Giezen, 2018). Historically, diagnosis of *Blastocystis* has been based on light microscopy of fecal smears or *in vitro* cultures. The organism has four morphological forms: vacuolar, granular, amoeboid, and cyst (Tan, 2008; Parija and Jeremiah, 2013). The lack of distinct morphological features had, in the past, blurred the extent of *Blastocystis* diversity. Based on the genetic heterogeneity of the small subunit ribosomal RNA (*SSU* rRNA), *Blastocystis* is currently divided into at least 28 subtypes (STs) consisting of ST1-ST17, ST21, and ST23-ST32, all of which have been found in mammalian and avian hosts and are likely separate species (Stensvold et al., 2012; Alfellani et al., 2013b; Zhao et al., 2017; Maloney et al., 2020, 2021a,b; Stensvold and Clark, 2020; Higuera et al., 2021). Several genetically distinct *Blastocystis* lineages have also been identified in amphibian, insect and reptilian hosts, however, these are not part of the subtyping nomenclature as yet (Yoshikawa et al., 2007, 2016).

Despite earlier assumptions, subtypes do not seem to be host specific. So far, ST1-ST9 and ST12 have been reported in humans along with a single instance of ST10, ST14, and ST16 (Stensvold and Clark, 2016; Khaled et al., 2020; Osorio-Pulgarin et al., 2021). The most frequently encountered subtypes in humans are ST1-ST4, with the latter being most often reported in Europe (Deng et al., 2019; Jiménez et al., 2019; Stensvold et al., 2020). Nonetheless, the subtypes reported in humans have also been found in non-human hosts. For example, ST1 and ST3 have been identified from pigs, while ST4 is dominant in rodents (Yoshikawa et al., 2004; Stensvold et al., 2009a; Alfellani et al., 2013a; Wang et al., 2018; Betts et al., 2021).

After more than a century of research, the pathogenicity of *Blastocystis* remains questionable. Its presence in sufferers of chronic gastrointestinal illnesses including irritable bowel syndrome and inflammatory bowel disease has led to speculations about possible links to these disease states (Dogruman-Al et al., 2009a; Tan et al., 2010; Poirier et al., 2012; Cifre et al., 2018; Kesuma et al., 2019; Peña et al., 2020; Shirvani et al., 2020). However, recent studies have increasingly shown that *Blastocystis* is a frequent and stable inhabitant in the gut of hosts without gastrointestinal symptoms (Scanlan et al., 2014; Mirjalali et al., 2017; Riabi et al., 2018; Yowang et al., 2018; Kataki et al., 2019; Lhotská et al., 2020; Padukone et al., 2020). In parallel, this protist has been linked with increased bacterial richness and diversity in the human gut (Audebert et al., 2016; Chabé et al., 2017; Laforest-Lapointe and Arrieta, 2018; Tito et al., 2019; Deng et al., 2021). Therefore, a plethora of researchers now consider *Blastocystis* as a commensal rather than a pathogen.

Understanding various aspects of *Blastocystis* epidemiology will contribute significantly toward determining its pathogenicity and/or virulence of the various subtypes. To that end, elucidating routes of transmission and contributions of various sources to these routes is essential. The human-to-human, zoonotic, and waterborne transmission routes have been explored in relation to *Blastocystis* prevalence (Eroglu and Koltas, 2010; Alfellani et al., 2013b; Maloney et al., 2019). Occurrence of certain subtypes in both human and other animal hosts has led to the hypothesis that these are subtypes of zoonotic potential. For instance, ST5, typically found in pigs, and ST6, ST7 typical subtypes of avian hosts, have also been found in humans that handle them extensively (Wang et al., 2014; Greige et al., 2018). Transmission of ST8 has also been noted between non-human primates and their human zookeepers (Stensvold et al., 2009a). Waterborne transmission of *Blastocystis* has been long recognized (Li et al., 2012; Andersen and Stensvold, 2016). For instance, ST1 was identified in the water supply of a rural community in central Thailand and schoolchildren that consumed it (Leelayoova et al., 2008) and in untreated drinking water in Peninsular Malaysia (Anuar et al., 2013). Nonetheless, only scant studies simultaneously consider the contribution of more than one source to *Blastocystis* transmission.

In general, investigating transmission dynamics requires conditions that allow for uninterrupted cycling of an organism in a community. As such, developing countries comprise ideal areas to undertake these types of approaches. Herein, we undertook a One Health approach to examine *Blastocystis* epidemiology in a rural community of northern Thailand. We collected samples from humans, other animals and the environment and screened them for presence of *Blastocystis*. Data were analyzed at singular and community levels. We identified water and soil as the primary contributing sources to *Blastocystis* transmission routes in this particular community. These findings provide a multi-layered understanding of the transmission dynamics (spreading and cycling) of this controversial protist.

## Materials and Methods

### Ethics Statement

The ethics committee of Mae Fah Luang University approved collection of human and animal samples used in this study (human license approval number REH60103 and animal license approval number AR01/62). Ethical rules were in accordance to the Declaration of Helsinki. Data were strictly anonymized and each sample was assigned an individual barcode.

### Study Area

**FIGURE 1:**
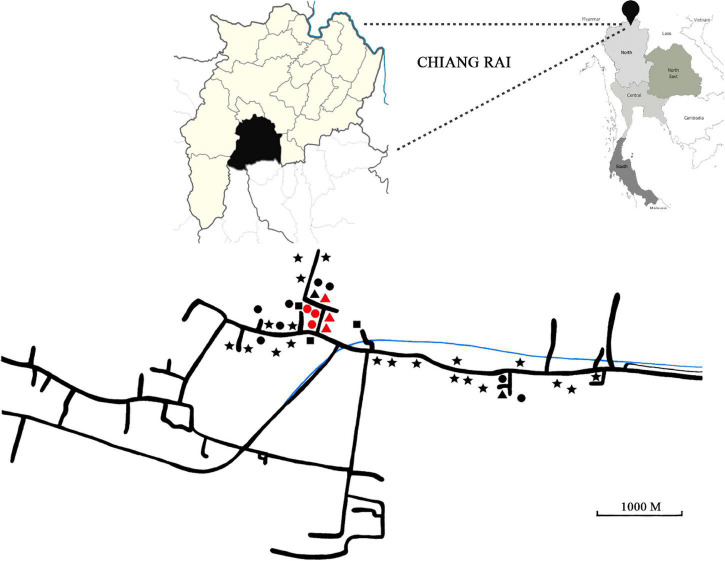
Top right panel: Map of Thailand. Black pin has been placed on Chiang Rai Province. Top left panel: Close-up of Chiang Rai Province (in pale yellow) and the district where sampling took place (in black). Bottom panel: Detail of area of collection used in this study. Geometrical shapes represent households. Stars: Only human stool was collected. Triangles: human stool, animal stool and water were collected. Squares: Human and animal stool was collected. Circles: Human stool and water were collected. Red shapes indicate households, where stool samples were collected from all members.

This study took place in a century-old rural community of 500 inhabitants in Chiang Rai Province, Thailand, between 2018 and 2019. The province is located in northern Thailand and borders Myanmar (Figure 1). The area of study is located across a river and villagers feed mainly on fish, vegetables and sticky rice. All residents are Thai nationals with no travel history of going abroad. There has been no immigration in the community for the last 20 years. The distance from the closest urban center is 20 km.

### Sample Collection

**FIGURE 2:**
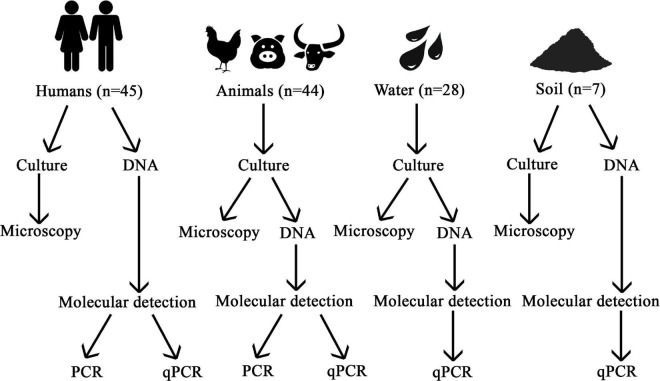
Flow chart of the methodology used in this study.

A summary of the methodology used is provided in Figure 2.

#### Human Fecal Samples

Fecal samples were collected from 45 Thai adults. Each participant was provided with a sterile sampling kit containing collection container, gauze and spatula. Volunteers did not suffer from gastrointestinal diseases and had no gastrointestinal symptoms at the time of sampling. Samples were collected from 39 households, six of which housed families. A family was defined as a group of at least two people living under the same roof.

#### Other Animal Fecal Samples

Fecal samples were collected from 44 animals including chickens, buffalo and pigs. These animals are representative of the livestock present in the community. Several stray dogs wander freely around the community and cannot be assigned to an owner, hence these were not sampled. The animals from which samples were obtained could be traced to specific households. Chickens (*n* = 34) were free-range and lived in tight proximity to the household, while buffalo (*n* = 4) and pigs (*n* = 6) were housed further away from the house. Animals did not have diarrhea or blood in their stool at the time of sampling.

#### Water Samples

Water in the area was surveyed to investigate the possibility of an environmental reservoir of *Blastocystis*. A total of 28 water samples were collected, 17 of which were from rain collection vessels (Supplementary Figure 1). These are cement containers (∼ 2 m in height) present in most houses. Most of the water comes from direct rain run-off from the roof of the house. A pipe directly connects the roof to the container. Cotton plugs serve as filters to catch leaves and wood debris. A lid rests over the containers most of the time. This water is used for drinking and cooking. The containers are washed once a year during the dry season. A tap is located at the bottom of each container. One liter of water was taken from the tap of each container. Water from all containers was turbid at visual inspection.

Three samples of 1 L each were collected from the single community supply water-dispensing machine (Supplementary Figure 2). The water comes from a waterfall, filtered and dispensed from the machine into 10 L containers. This water is used for drinking and cooking. The containers are washed with pressurized water regularly. We sampled three of those containers.

Two samples of 1 L each were collected from the water treatment facility, from which water is distributed to every household through pipes (Supplementary Figure 3). The water is taken directly from the river and occasionally treated with chlorine. This water is used for bathing and watering plants (edible and non-edible).

One sample was collected from the community water tower (Supplementary Figure 3C). The water from the tower comes from the water treatment facility. The bottom of each tower is lined with layers of sand and gravel, which serve as additional filters. Villagers can get their water through a tap located at the bottom of the tower. The water is used mostly for cooking and bathing and rarely for drinking. A 1 L sample was taken from the tap and the water was turbid at visual inspection.

Two samples of 1 L each were collected from the river stream, which is densely grown with morning glory plants (*Ipomoea aquatica Forssk*) and eaten raw or cooked (Supplementary Figure 4A). At the time of collection, water depth was 20 cm. Water was collected from the middle of the stream and was very turbid at visual inspection.

A single sample was collected from an artificial pond with soil sediment. The pond is used for fish farming (Supplementary Figure 4B). A 1 L sample was taken from the shallow end of the pond. Sample was very turbid at visual inspection.

Two samples were taken from a cement container, which is used for short term holding of live fish and amphibians (Supplementary Figure 4C). Occasionally, the water from the pond and the cement container is used for watering gardens. A 1 L sample was collected and was slightly turbid at visual inspection.

#### Soil Samples

Seven soil samples were collected from a depth of no more than 5 cm using sterile spoons. Each sample consisted of 2–3 g of soil. Four of these came from four separate vegetable gardens (Supplementary Figures 5A–D). Three of the gardens were field plots, while one comprised of pots. One soil sample came from an ephemeral stream, where the local herb Plu Kaow grows (*Houttuynia cordata Thunb*). Villagers use this herb extensively (raw or cooked) for vegetable side dishes accompanying raw meat. The stream was void of water, but muddy at the time of collection. One sample was also gathered from river sediment. One soil sample was picked from the riverbank. Both the river and the riverbank are overgrown with morning glory (Supplementary Figure 6).

### *Blastocystis* Cultures

For human and other animal fecal samples, approximately 200 mg of freshly collected feces were placed in LYSGM (Diamond, 1982) containing 10% horse serum. Water samples were left to sit for 3 h on a flat surface. Subsequently, 2–4 mL was taken from the bottom of each sample and placed in LYSGM. Soil samples were thoroughly mixed and 100 mg placed in LYSGM. Tubes were incubated at 37°C for 48–72 h and screened for *Blastocystis* using light microscopy.

### Genomic DNA Extraction

#### Human and Other Animal Fecal Samples

In the case of human samples, DNA was extracted from feces using 200 mg. DNA from animal samples was extracted prior to the first passage of culture using 250 mL of sediment from each sample. The Qiagen DNA stool minikit (Qiagen, Hilden, Germany) was used according to manufacturer’s protocol.

#### Water Samples

DNA was extracted from 250 mL of culture sediment using AccuPrep^®^ Genomic DNA Extraction Kit following the manufacturer’s protocol.

#### Soil Samples

DNA from soil was directly extracted from 200 mg of soil using PowerSoil^®^ DNA Isolation Kit (Carlsbad, CA United States) according to manufacturer’s protocol.

### *Blastocystis* Detection

Three approaches were used to detect *Blastocystis* from human samples: microscopy following culturing in LYSGM, conventional PCR and qPCR (Figure 2). For the rest of the samples only microscopy and qPCR were used.

### Polymerase Chain Reaction and Sequencing

The broad specificity primer pair RD3 5′-GGGATCCTGA TCCTTCCGCAGGTTCACCTAC-3′ and RD5 5′-GGAAGC TTATCTGGTTGATCCTGCCAGTA-3′ (Clark, 1997) was used for the first PCR reactions with the following conditions: initial denaturation for 3 min at 94°C, 35 cycles at 94°C for 1 min, annealing 60°C for 1 min, and extension at 72°C for 100 s, with a final elongation step at 72°C for 7 min. A 600 bp fragment of *SSU* rRNA gene region, which is also the barcode region of *Blastocystis* was amplified with a second nested PCR. The PCR reaction was carried out by using the forward BsRD5F (5′-ATCTGGTTGATCCTGCCAGT-3′) and reverse BhRDr9R (5′-GAGCTTTTTAACTGCAACAACG-3′) barcoding primers (Scicluna et al., 2006). The PCR conditions consisted of initial denaturation for 3 min at 94°C, 35 cycles at 94°C for 1 min, annealing 60°C for 1 min, and extension at 72°C for 100 s, with a final elongation step at 72°C for 10 min. Positive and negative controls were included with each batch of samples analyzed.

### Quantitative Polymerase Chain Reaction

*Blastocystis* prevalence was assessed using qPCR to amplify a 330 bp fragment of the *SSU* rRNA gene. The qPCR reactions mixture were performed in 10 μL reaction mixture volume with 3 μL of water, 4 μL SensiFAST™ SYBR No-ROX Kit (BIOLINE, United Kingdom), 0.5 μL of each forward (BL18SPPF1; 5′-AGTAGTCATACGCTCGTCTCAAA-3′) and reverse (BL18SR2PP; 5′-TCTTCGTTACCCGTTACTGC-3′) *Blastocystis*-specific primer and 2 μL of genomic DNA. The qPCR amplification conditions were as previously described (Poirier et al., 2011). Reactions were run in 96-well plates in a CFX96 Touch™ Real-Time PCR Detection System (Bio-Rad, United States). Positive and negative controls were used in each qPCR run together with all samples. Each type of sample was run separately to avoid cross-contamination. For example, soil sample experiments were executed on separate plates and on separate days from water, human and animal samples.

### Amplicon Purification and Sequencing

All positive PCR and qPCR products were purified using the GeneJET Gel Extraction Kit (Thermo Fisher Scientific; Wardmedic, Thailand) according to manufacturer’s instructions and sequenced at U2Bio (Korea).

### Cloning

Twenty-one samples showing long stretches of indistinguishable peaks were cloned, six of which were PCR products and 15 qPCR. Five samples were human, two buffalo, two pig, four chickens, four water and four soil. 1.5 μL of amplicon was used with the pGEM-T easy vector system I (Promega, Madison, WI, United States) following previously published cloning protocols (Betts et al., 2018). Up to five colonies per transformation were screened.

### Phylogenetic Analysis

The chromatogram quality of raw reads was checked using the chromatogram visualization software 4Peaks. Ambiguous bases at the ends of the reads were removed. The new sequences were then used as queries to perform blast searches against the NCBI nr database. Sequences of *SSU* rRNA spanning the spectrum of *Blastocystis* diversity were downloaded and aligned using mafft v. 7.394 (Katoh and Toh, 2010). Ambiguous positions were removed using trimal v. 1.4 and gappyout option (Capella-Gutierrez et al., 2009). The final trimmed alignment consisted of 250 taxa and 1497 sites. Maximum likelihood (ML) analysis was performed in CIPRES Science Gateway (^1^ Miller et al., 2010) using RAxML-HPC2 on XSEDE (Stamatakis, 2006). Bootstrap support was computed from 1,000 pseudoreplicates.

## Results

### Human Demographic Data

A total of 45 human volunteers participated in this study (31% male, *n* = 14 and 69% female, *n* = 31), with mean age of 59.1 ± 8.5 years (median = 60).

### Comparison of Microscopy and Molecular Methods in Human Stool Samples

The prevalence of *Blastocystis* in all human stool samples was observed using morphology and molecular techniques (Table 1). All samples were cultured in LYSGM and of these, 9% (4/45) were microscopy-positive for *Blastocystis*. Using conventional PCR, 49% (22/45) of samples were positive, while the number increased to 73% (33/45), when using qPCR. All microscopy-positive samples were also positive using molecular detection. Eleven PCR samples were false positive by Sanger sequencing (plants and fungi rather than *Blastocystis*), thus PCR positivity rate of *Blastocystis* confirmed by sequencing was 27% (12/45). One qPCR product was false positive by Sanger sequencing (Fungi; not included in the prevalence calculation). The prevalence rates reported are based only on samples that have been sequenced and are indeed verified as *Blastocystis*.

**TABLE 1:** Comparison of microscopy and molecular methods.

Methods	Prevalence	
	Positive	Negative
**Morphology**		
Light microscopy	4 (8.89%)	41 (91.11%)
**Molecular**		
Polymerase chain reaction (PCR)	12 (26.67%)	33 (73.33%)
quantitative Polymerase chain reaction (qPCR)	33 (73.33%)	12 (26.67%)

### Prevalence and Diversity of *Blastocystis* in Animal and Environmental Samples

Forty-four fecal samples were collected from animals as follows: chickens (*n* = 34), pigs (*n* = 6) and buffalo (*n* = 4). All animal samples were cultured in LYSGM. Using microscopy, 65% (22/45) of chicken cultures were positive, while no *Blastocystis* was observed in pig and buffalo cultures. Using qPCR and subsequent sequencing, the prevalence of *Blastocystis* was 100% in chickens, pigs and buffalo. Overall, 28 samples of water and seven samples of soil were cultured and surveyed for *Blastocystis*. Two water and one soil sample were false positives for Cercozoa and bacteria and were not considered for further analysis. Prevalence using qPCR was 93% (26/28) for water and 86% (6/7) for soil. The reported prevalence rates are based solely on samples that have been sequenced and verified as *Blastocystis*.

Of the PCR and qPCR *Blastocystis* positive samples that were sequenced, 21 were cloned: Cloning yielded 62 clones, of which 17 were from human fecal samples, six from buffalo, nine from pig, 14 from chicken, nine from water and seven from soil (Supplementary Material 2). The following subtypes (STs) were identified: ST1, ST2, ST3, ST4, ST5, ST6, ST7, ST10, ST23, and ST26 (Table 2). Nine sequences could not be subtyped either because of poor quality or short length. Eight of the identified subtypes were found in humans. The dominant subtype was ST23 (12/33, 36%), followed by ST10 (6/33, 18%), ST1 (5/33, 15%), ST3 (2/33, 6%) and a single occurrence of ST2 (1/33, 3%), ST4 (1/33, 3%), ST5 (1/33, 3%), and ST7 (1/33, 3%). Chickens carried ST6 (2/33, 3%) and ST7 (31/33, 94%), pigs ST7 (6/6, 100%), and buffalo ST7 (2/4, 50%) and ST23 (2/4, 100%). Subtype 1 (5/26, 19%), ST3 (13/26, 50%), ST6 (1/26, 4%), ST7 (1/26, 4%), ST23 (1/26, 4%), and ST26 (2/26, 8%) were detected in water, whereas in the soil samples ST1 (1/6, 17%), ST3 (2/6, 33%), ST7 (2/6, 33%), ST23 (3/6, 50%), and ST26 (2/6, 33%) were found. Three humans carried both ST10 and ST23. Within subtypes, multiple genetically diverse strains were present in ST7, while ST1, ST3, ST5, and ST6 sequences were much more genetically similar (data not shown).

**TABLE 2:** Prevalence and subtypes of *Blastocystis* in human, animal, water and soil samples.

Source	B + ve	ST1	ST2	ST3	ST4	ST5	ST6	ST7	ST10	ST23	ST26	UNK
Human	33	5	1	2	1	1	–	1	6	12	–	1
Chicken	33	–	–	–	–	–	2	31	–	–	–	–
Pig	6	–	–	–	–	–	–	6	–	–	–	–
Buffalo	4	–	–	–	–	–	–	2	–	2	–	–
Water	26	5	–	13	–	–	1	1	1	1	2	2
Soil	6	1		2	–	–	–	2	–	3	1	–
**Total**	**108**	**11**	**1**	**17**	**1**	**7**	**3**	**37**	**7**	**18**	**3**	**3**

A detailed account of all newly generated sequences is provided in Supplementary Material 2. All 149 sequences generated in this study have been submitted to GenBank under accession numbers OL351649–OL351797.

### Phylogenetic Analysis

**FIGURE 3:**
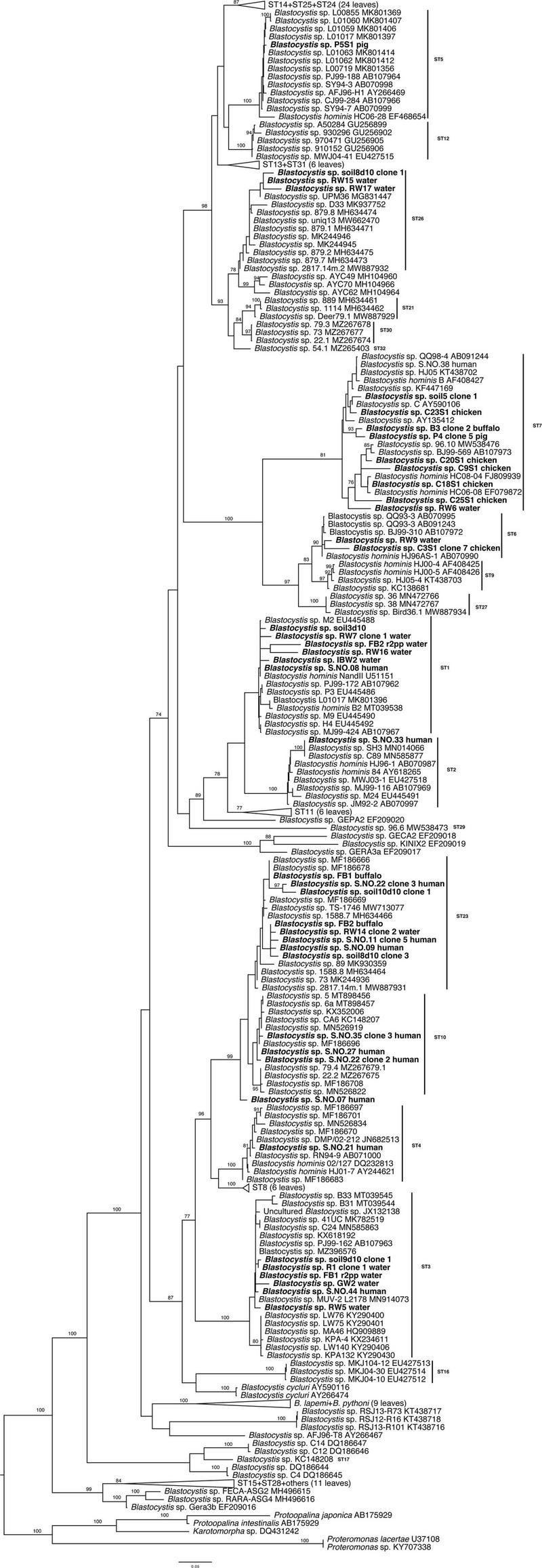
Maximum likelihood phylogenetic tree inferred from 250 taxa and 1,497 sites of the *SSU* rRNA gene. The tree is artificially rooted to *Proteromonas*, *Protoopalina*, and *Karotomorpha* sequences. Newly generated sequences are in bold. Numerical values indicate bootstrap support. Only values above 70% are depicted.

All *Blastocystis* sequences grouped together with maximum bootstrap support (BS) (Figure 3). Subtypes 15 and 28 along with sequences from ectothermic hosts placed in the base of the tree in agreement with previous studies (Higuera et al., 2021). Subtype 5, ST12, ST13, ST14, ST24, and ST25 formed a clade sister to the clade formed by ST26, ST21, ST30, and ST32. Distinct clades of subtypes were as follows: ST6 and ST7; ST1, ST2, and ST11; ST23 and ST10; and ST4 and ST8. Newly generated sequences placed within clades consisting of known subtypes with the exception of the human origin sequence S.NO.07. Notably, the positions of the new sequences placing with ST10 and ST23 are not entirely robust, suggesting that perhaps these are new, closely related subtypes. Nonetheless, without full length sequences further conclusions cannot be drawn (this is currently under investigation).

### Transmission Dynamics

#### Household Level

Samples were collected from a total of 39 households. In most cases, a single individual per household was sampled, with the exception of six households where all samples from all individuals were collected (Figure 1). Of those, five households were found positive for *Blastocystis.* In two of them, only the male occupant was positive. In the other three households, both occupants were positive, but carried different subtypes.

#### Farm Animal Ownership Level

Of the 39 sampled households, eight of them had animals (seven with chickens and one with buffalo). *Blastocystis* was found in six of these households and there was no subtype sharing between animal and human hosts (Table 3).

**TABLE 3:** Prevalence of *Blastocystis* in animals and their animal-keepers.

Household	Animals	*Blastocystis* in humans	*Blastocystis* in animals
1	Chicken	Negative (*n* = 2)	ST7 (*n* = 10)
2	Chicken	ST1 (*n* = 1) ST10 (*n* = 1)	ST7 (*n* = 5)
3	Chicken	ST23 (n-1) unknown (*n* = 1)	ST7 (*n* = 5)
4	Chicken	ST23 (*n* = 1)	ST7 (*n* = 3)
5	Chicken	ST3 (*n* = 1)	ST7 (*n* = 4)
6	Chicken	Unknown (*n* = 1)	ST7 (*n* = 3)
7	Buffalo	ST4 (*n* = 1)	ST23, ST7 (*n* = 4)
8	Chicken	Negative (*n* = 1)	ST7 (*n* = 4)

#### Environmental Level

Of the 39 sampled households, 16 were sampled for water and six for soil, all of which were positive for *Blastocystis*. There was subtype overlap between water and humans in one household (ST3).

#### Community Level

Out of the 108 *Blastocystis* positive samples, 33 (31%) were from humans, 43 (40%) from animals, 26 (24%) from water and 6 (6%) from soil. Subtype 2 and ST4 were identified only in humans, whereas ST26 was only found in the environmental samples (both soil and water). Subtype 7 was the most broadly distributed as it was found in humans, pig, buffalo and chicken, but also in soil and water. Subtype 1, ST3, ST7, ST10, and ST23 were found in human and environmental samples. No subtype was exclusively shared by only humans and other animals. Subtype 6 was the only one shared between animals and the environment.

## Discussion

The study took place in a century old isolated rural community in northern Thailand comprising approximately 500 people. Inhabitants live in very close proximity to their animals, primarily chickens and secondarily buffalo and pigs. Part of the community’s water supply comes from the river that runs through it. The river also provides a major food source for the villagers, as fish constitutes the primary protein source of the community, along with vegetables (which also grow inside the river and the river bank) and locally farmed sticky rice. The increased influence of westernized diet noted in urban centers of Thailand has a minor impact in this community. Collectively, the small population, distance from urban centers, unique gastronomy (minimal effect from westernization) and the general lifestyle make this particular community ideal for local One Health approaches. Herein, we used *Blastocystis*, a microbial eukaryote of controversial pathogenicity, to obtain a comprehensive view of its transmission dynamics.

*Blastocystis* is the most frequently encountered intestinal protist of metazoans with most studies focusing on either its prevalence in humans, other animals and/or the environment. Nonetheless, only very few investigations explore the organism’s transmission dynamics using a tripartite approach, whereby all of the aforementioned factors are considered collectively. In order to understand the role of this organism in health and disease it is essential to determine its occurrence simultaneously in human and non-human hosts and environments.

In humans, the prevalence of *Blastocystis* has been frequently reported in those with and without gastrointestinal symptoms (Dogruman-Al et al., 2009b; Scanlan et al., 2014; Yowang et al., 2018; Kataki et al., 2019; Lhotská et al., 2020; Padukone et al., 2020). Overall prevalence of *Blastocystis* might vary due to sampling population, region and detection method (Stensvold et al., 2009b; Tan et al., 2010; Alfellani et al., 2013a; Anuar et al., 2013; Clark et al., 2013). Herein, the prevalence of *Blastocystis* in asymptomatic human hosts was 73%, in asymptomatic non-human hosts 100% and in environmental samples 91%. We used microscopy and molecular methods to determine presence of *Blastocystis*. The most sensitive detection method was qPCR matching previous studies (Poirier et al., 2011; Stensvold and Nielsen, 2012; Stensvold et al., 2012). After sequencing all positive samples, a broad diversity of subtypes (STs) was detected: ST1, ST2, ST3, ST4, ST5, ST6, ST7, ST10, ST23, and a potential new subtype. Subtype 10 was detected in six human volunteers. The subtype has been previously found in two Senegalese children (Khaled et al., 2020), but it is a typical cattle subtype (Cian et al., 2017; Zhu et al., 2017; Masuda et al., 2018; Wang et al., 2018). To our great surprise, we found ST23 in 12 human samples making it the dominant subtype in this host. So far, ST23 has only been identified in ruminants. The occurrence of ST10 and ST23 in several adults in an Asian country raises questions regarding the host range and transmission dynamics of *Blastocystis* subtypes.

The following transmission routes have been widely discussed for *Blastocystis*: human-to-human, animal-to-human and environment-to-human. The former mode of transmission has been speculated to occur via the fecal-oral route much like other common gastrointestinal parasites. Herein, investigation of individuals within households showed no subtype sharing and there was even an instance of co-habiting individuals, whereby one was *Blastocystis* positive and another negative. This finding matches previous recent reports derived from family units elsewhere (Scanlan et al., 2016; Lhotská et al., 2020).

We also aimed to look at the animal-to-human transmission route. Previous studies have suggested that specific subtypes are zoonotic (Parkar et al., 2010; Alfellani et al., 2013b; Wang et al., 2014). For instance, ST5 has been proposed as potentially zoonotic from pigs (Yan et al., 2007; Wang et al., 2014) and *Blastocystis* ST6 and ST7 from poultry (Ramírez et al., 2014; Cian et al., 2017; Greige et al., 2018; Udonsom et al., 2018). Subtype 1, ST7, ST10, and ST23 were found in both human and animal hosts in the studied area giving the impression of zoonotic transmission. However, when looking at a fine-scale level there was no sharing of subtypes between animals and their respective owners. Collective consideration of the evidence points toward the source of *Blastocystis* in this specific community being elsewhere.

This prompted us to look at the two most commonly encountered environmental sources in the community: water and soil. Water contamination has been speculated as a risk factor to acquire *Blastocystis*. However, only few studies have looked at presence of *Blastocystis* in both water and humans that use it and even fewer have employed subtyping to examine overlap between the two (Leelayoova et al., 2008; Angelici et al., 2018; Pawestri et al., 2021). *Blastocystis* has been detected in drinking water (Leelayoova et al., 2008), tap water (Eroglu and Koltas, 2010), rain water tanks (Noradilah et al., 2017; Waters et al., 2019), bodies of freshwater (Ithoi et al., 2011; Khalifa et al., 2014), drinking water treatment facilities (Richard et al., 2016) and waste water (Suresh et al., 2005; Banaticla and Rivera, 2011; Stensvold et al., 2020). Herein, *Blastocystis* ST1 and ST3 were detected in community supply water, while ST1, ST3, ST5, ST6, ST7, ST10, ST23, and ST26 were found in rain collection vessels. Both these sources comprise the drinking water of this community. The rain collection vessels contain water that is filtered for large debris, but the water is untreated and is consumed unboiled (Li et al., 2007; Leelayoova et al., 2008; Anuar et al., 2013; Wongthamarin et al., 2018; Waters et al., 2019). The community supply water is filtered and occasionally treated. Given the exposed nature of the community water, various wildlife animal hosts harboring a range of subtypes (known and unknown) can easily access it. Thus, presence of the organism in these two sources could be due to a combination of factors including contamination by animal droppings and/or substandard management (i.e., filtration and chlorine usage). Water in the vessels is also used to wash vegetables and tubers hence transfer of cysts of a variety of subtypes could occur this way. Indeed *Blastocystis* has been previously found in vegetables (Al Nahhas and Aboualchamat, 2020; Li et al., 2020). Through fine-scale analysis we identified a case of ST3 in humans overlapping with the subtypes found in their rain collection vessels. Presence of *Blastocystis* in an environment, where there is continuous circulation of oxygen supported recently raised hypotheses that this previously considered strictly anaerobic organism tolerates oxygen (Tsaousis et al., 2012, 2018). Thus, future studies should aim toward investigating additional environments including extreme habitats for the presence of *Blastocystis*.

**FIGURE 4:**
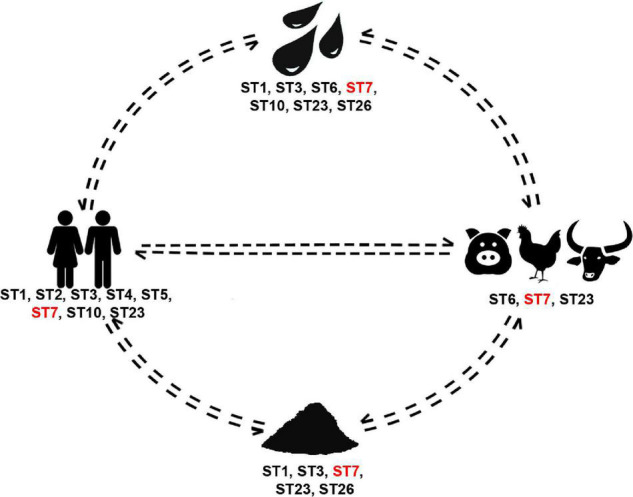
*Blastocystis* subtype cycling in the rural community studied herein. Subtypes present in all sources are in red font.

To that end, we broadened our approach and also explored occurrence of *Blastocystis* in soil. Most collected soil samples were positive for the organism, while ST1, ST3, ST7, ST23, and ST26 were identified. To our knowledge this is the first report of this protist being recorded in natural soil. The presence of *Blastocystis* in the soil could be due to extensive use of animal excrement and intestinal contents (especially from fish), which are typically utilized as garden fertilizer in the community. Nonetheless, while sampling, care was taken to collect from gardens that had not been recently fertilized. Moreover, wildlife hosts roaming the community could also shed *Blastocystis*. This finding suggests a new route of transmission that has been previously overlooked. In that vein, we propose that soil should not only be checked for presence of the organism in future studies, but that it should also be included along with water as a transmission route in the life cycle of *Blastocystis* (Figure 4).

Comparison of sequences found in different hosts and environments indicated that highly similar strains of ST1 and ST3 are circulating in the community. This suggests a shared *Blastocystis* transmission cycle among humans, animals and the environment for these subtypes. In contrast, ST7 showed an extraordinary amount of diversity with multiple strains distributed within and between hosts and the environment. This indicates that the full extent of ST7 genetic diversity and host range in the community has yet to be captured. Nonetheless a cluster of highly similar strains was found in chicken, pig and buffalo suggesting transmission among the three hosts.

This study has revealed no clear patterns of direct transmission between human-to-human or animal-to-human in this community (Figure 4). Instead, it points out to the existence of multiple independent routes of transmission. Previous efforts investigating *Blastocystis* sources of transmission have been geared toward dissecting dipartite relationships (i.e., animal-to-human or environment-to-human). Results from these studies have enhanced our understanding of the organism and its epidemiology. Nonetheless, they frequently only provide pieces of the overall picture, which remains fragmentary. Here, we have provided a step forward toward integrating a One Health approach to *Blastocystis* by considering both living and non-living sources. In this community, environmental sources comprise the reservoir of *Blastocystis* supplying a multitude of subtypes that circulate in both human and non-human hosts. Our study is pioneer in that we investigated a rural area, while taking into account the community structure and environmental factors toward understanding *Blastocystis* circulation.

Limited sample size does pose a limitation in our study. Specifically, the sample size was low, in particular samples from various animal hosts including stray animals and wildlife. Thus transmission cycles between and within hosts and the environment cannot be precisely deduced at this time.

Moving forward, additional communities both rural and urban should be explored under the One Health umbrella to determine whether similar patterns occur. Using the same approach in a temporal context, future studies should also investigate, whether *Blastocystis* and its various subtypes are true colonizers or passengers. Finally, supplementing One Health-based studies with culturomics and microbiome (pathogenic and non-pathogenic residents of the gut) and metabolome investigations will contribute significantly in uncovering the true roles of *Blastocystis* in gut health and disease.

## Data Availability Statement

The datasets presented in this study can be found in online repositories. The names of the repository/repositories and accession number(s) can be found in the article/Supplementary Material.

## Ethics Statement

The studies involving human participants were reviewed and approved by the Ethics Committee of Mae Fah Luang University. The patients/participants provided their written informed consent to participate in this study. The animal study was reviewed and approved by the Ethics Committee of Mae Fah Luang University. Written informed consent for participation was not obtained from the owners because only fecal samples were obtained, there was no invasive procedure involved.

## Author Contributions

VJ: fieldwork, methodology, conceptualization, investigation, and draft writing. SM: methodology. SP: resources and methodology. AT: supervision, validation, and final draft. EG: funding acquisition, project administration, supervision, validation, data curation, and final draft. All authors have read and approved the submitted version of the manuscript.

## Conflict of Interest

The authors declare that the research was conducted in the absence of any commercial or financial relationships that could be construed as a potential conflict of interest.

## Publisher’s Note

All claims expressed in this article are solely those of the authors and do not necessarily represent those of their affiliated organizations, or those of the publisher, the editors and the reviewers. Any product that may be evaluated in this article, or claim that may be made by its manufacturer, is not guaranteed or endorsed by the publisher.

## Footnotes

1
https://www.phylo.org/portal2/login!input.action

## Supplementary Material

Supplementary Material 1 Includes Supplementary Figures 1–6, which are images from the collection sites.

Supplementary Material 2 Includes a detailed account of all newly generated sequences.

## References

[B1] Al Nahhas S. Aboualchamat G. (2020). Investigation of parasitic contamination of salad vegetables sold by street vendors in city markets in Damascus, Syria. *Food Waterborne Parasitol*. 21:e00090. 10.1016/j.fawpar.2020.e00090 33241130 PMC7672268

[B2] Alfellani M. A. Taner-Mulla D. Jacob A. S. Imeede C. A. Yoshikawa H. Stensvold C. R. (2013b). Genetic diversity of *Blastocystis* in livestock and zoo animals. *Protist* 164 497–509. 10.1016/J.PROTIS.2013.05.003 23770574

[B3] Alfellani M. A. Stensvold C. R. Vidal-Lapiedra A. Onuoha E. S. U. Fagbenro-Beyioku A. F. Clark C. G. (2013a). Variable geographic distribution of *Blastocystis* subtypes and its potential implications. *Acta Trop.* 126 11–18. 10.1016/J.ACTATROPICA.2012.12.011 23290980

[B4] Andersen L. O. B. Stensvold R. C. (2016). *Blastocystis* in health and disease: are we moving from a clinical to a public health perspective? *J. Clin. Microbiol.* 54 524–528. 10.1128/JCM.02520-15 26677249 PMC4767957

[B5] Angelici M. C. Nardis C. Scarpelli R. Ade P. (2018). *Blastocystis hominis* transmission by non-potable water: a case report in Italy. *New Microb.* 41 173–177. 29498738

[B6] Anuar T. S. Ghani M. K. A. Azreen S. N. Salleh F. M. Moktar N. (2013). *Blastocystis* infection in Malaysia: evidence of waterborne and human-to-human transmissions among the Proto-Malay, Negrito and Senoi tribes of Orang Asli. *Parasit. Vectors* 6:40. 10.1186/1756-3305-6-40 23433099 PMC3584727

[B7] Audebert C. Even G. Cian A. Loywick A. Merlin S. Viscogliosi E. (2016). Colonization with the enteric protozoa *Blastocystis* is associated with increased diversity of human gut bacterial microbiota. *Sci. Rep*. 6:25255. 10.1038/srep25255 27147260 PMC4857090

[B8] Banaticla J. E. G. Rivera W. L. (2011). Detection and subtype identification of *Blastocystis* isolates from wastewater samples in the Philippines. *J. Water Health* 9 128–137. 10.2166/wh.2010.127 21301121

[B9] Beghini F. Pasolli E. Truong T. D. Putignani L. Cacciò S. M. Segata N. (2017). Large-scale comparative metagenomics of *Blastocystis*, a common member of the human gut microbiome. *ISME J.* 11 2848–2863. 10.1038/ismej.2017.139 28837129 PMC5702742

[B10] Betts E. L. Gentekaki E. Thomasz A. Breakell V. Carpenter A. I. Tsaousis A. D. (2018). Genetic diversity of *Blastocystis* in non-primate animals. *Parasitology* 145, 1228–1234. 10.1017/S0031182017002347 29338807 PMC5912512

[B11] Betts E. L. Hoque S. Torbe L. Bailey J. R. Ryan H. Toller K. (2021). Parasites, drugs and captivity: blastocystis-microbiome associations in captive water voles. *Biology* 6:457. 10.3390/biology10060457 34067374 PMC8224621

[B12] Capella-Gutierrez S. Silla-Martinez J. M. Gabaldon T. (2009). Trimal: a tool for automated alignment trimming in large-scale phylogenetic analyses. *Bioinformatics (Oxford, England)* 25 1972–1973. 10.1093/bioinformatics/btp348 19505945 PMC2712344

[B13] Chabé M. Lokmer A. Ségurel L. (2017). Gut Protozoa: friends or foes of the human gut microbiota? *Trends Parasitol.* 33 925–934. 10.1016/J.PT.2017.08.005 28870496

[B14] Cian A. El Safadi D. Osman M. Moriniere R. Gantois N. Benamrouz-Vanneste S. (2017). Molecular epidemiology of *Blastocystis* sp. in various animal groups from two French zoos and evaluation of potential zoonotic risk. *PLoS One* 12:e0169659. 10.1371/journal.pone.0169659 28060901 PMC5217969

[B15] Cifre S. Gozalbo M. Ortiz V. Soriano J. M. Merino J. F. Trelis M. (2018). *Blastocystis* subtypes and their association with Irritable Bowel Syndrome. *Med. Hypotheses* 116 4–9. 10.1016/J.MEHY.2018.04.006 29857906

[B16] Clark C. G. (1997). Extensive genetic diversity in *Blastocystis hominis*. *Mol. Biochem. Parasitol.* 87 79–83. 10.1016/s0166-6851(97)00046-7 9233675

[B17] Clark C. G. van der Giezen M. Alfellani M. A. Stensvold C. R. (2013). Recent developments in *Blastocystis* research. *Adv. Parasitol.* 82 1–32. 10.1016/B978-0-12-407706-5.00001-0 23548084

[B18] Deng L. Chai Y. Zhou Z. Liu H. Zhong Z. Hu Y. (2019). Epidemiology of *Blastocystis* sp. infection in China: a systematic review. *Parasite* 26:41. 10.1051/parasite/2019042 31309925 PMC6632114

[B19] Deng L. Wojciech L. Gascoigne N. R. J. Peng G. Tan K. S. W. (2021). New insights into the interactions between *Blastocystis*, the gut microbiota, and host immunity. *PLoS Pathog.* 17:e1009253. 10.1371/journal.ppat.1009253 33630979 PMC7906322

[B20] Diamond L. S. (1982). A new liquid medium for xenic cultivation of *Entamoeba histolytica* and other lumen-dwelling protozoa. *J. Parasitol.* 68 958–959. 6290631

[B21] Dogruman-Al F. Kustimur S. Yoshikawa H. Tuncer C. Simsek Z. Tanyuksel M. (2009a). *Blastocystis* subtypes in irritable bowel syndrome and inflammatory bowel disease in Ankara, Turkey. *Mem. Inst. Oswaldo Cruz* 104 724–727. 10.1590/s0074-02762009000500011 19820833

[B22] Dogruman-Al F. Yoshikawa H. Kustimur S. Balaban N. (2009b). PCR-based subtyping of *Blastocystis* isolates from symptomatic and asymptomatic individuals in a major hospital in Ankara, Turkey. *Parasitol. Res.* 106 263–268. 10.1007/s00436-009-1658-8 19847459

[B23] Eroglu F. Koltas I. S. (2010). Evaluation of the transmission mode of B. *hominis* by using PCR method. *Parasitol. Res.* 107 841–845. 10.1007/s00436-010-1937-4 20544220

[B24] Greige S. Safadi D. Bécu N. Gantois N. Pereira B. Chabé M. (2018). Prevalence and subtype distribution of *Blastocystis* sp. isolates from poultry in Lebanon and evidence of zoonotic potential. *Parasit. Vectors* 11:389. 10.1186/s13071-018-2975-5 29973261 PMC6030734

[B25] Higuera A. Herrera G. Jimenez P. Garcia-Corresor D. Pulido-Medellin M. Bulla-Castaneda D. M. (2021). Identification of multiple *Blastocystis* subtypes in domestic animals from Columbia using amplicon-based next generation sequencing. *Front. Vet. Sci*. 8:732129. 10.3389/fvets.2021.732129 34504891 PMC8421793

[B26] Ithoi I. Jali A. Mak J. W. Yusoff W. Sulaiman W. Mahmud R. (2011). Occurrence of *Blastocystis* in water of two rivers from recreational areas in Malaysia. *J. Parasitol. Res.* 2011:123916. 10.1155/2011/123916 21772980 PMC3135064

[B27] Jiménez P. A. Jaimes J. E. Ramírez J. D. (2019). A summary of *Blastocystis* subtypes in North and South America. *Parasit. Vectors* 12:376. 10.1186/s13071-019-3641-2 31358042 PMC6664531

[B28] Kataki M. M. Tavalla M. Beiromvand M. (2019). Higher prevalence of *Blastocystis hominis* in healthy individuals than patients with gastrointestinal symptoms from Ahvaz, southwestern Iran. *Comp. Immunol. Microbiol. Infect. Dis.* 65 160–164. 10.1016/j.cimid.2019.05.018 31300108

[B29] Katoh K. Toh H. (2010). Parallelization of the MAFFT multiple sequence alignment program. *Bioinformatics (Oxford, England)* 26 1899–1900. 10.1093/bioinformatics/btq224 20427515 PMC2905546

[B30] Kesuma Y. Firmansyah A. Bardosono S. Sari I. P. Kurniawan A. (2019). *Blastocystis* ST-1 is associated with Irritable Bowel Syndrome-diarrhoea (IBS-D) in Indonesian adolescences. *Parasite Epidemiol. Control* 6:e00112. 10.1016/j.parepi.2019.e00112 31528737 PMC6742775

[B31] Khaled S. Gantois N. Ly A. T. Senghor S. Even G. Dautel E. (2020). Prevalence and subtype distribution of *Blastocystis* sp. in Senegalese school children. *Microorganisms* 8:1408. 10.3390/microorganisms8091408 32932661 PMC7564003

[B32] Khalifa R. Ahmad A. K. Abdel-Hafeez E. H. Mosllem F. A. (2014). Present status of protozoan pathogens causing water-borne disease in northern part of El-Minia Governorate. *Egypt. J. Egypt. Soc. Parasitol.* 44 559–566. 10.21608/jesp.2014.90143 25643498

[B33] Laforest-Lapointe I. Arrieta M.-C. (2018). Microbial eukaryotes: a missing link in gut microbiome studies. *MSystems* 3 e201–e217. 10.1128/mSystems.00201-17 29556538 PMC5850078

[B34] Leelayoova S. Siripattanapipong S. Thathaisong U. Naaglor T. Taamasri P. Piyaraj P. (2008). Drinking Water: a possible source of *Blastocystis* spp. subtype 1 infection in schoolchildren of a rural community in central Thailand. *Am. J. Trop. Med. Hyg. Am. J. Trop. Med. Hyg.* 79 401–406. 10.4269/ajtmh.2008.79.401 18784233

[B35] Lhotská Z. Jirků M. Hložková O. Brožová K. Jirsová D. Stensvold C. R. (2020). A study on the prevalence and subtype diversity of the intestinal protist *Blastocystis* sp. in a gut-healthy human population in the Czech Republic. *Front. Cell. Infect. Microbiol.* 10:544335. 10.3389/fcimb.2020.544335 33123491 PMC7573152

[B36] Li J. Wang Z. Karim R. Zhang L. (2020). Detection of human intestinal protozoan parasites in vegetables and fruits: a review. *Parasit. Vectors* 13:380. 10.1186/s13071-020-04255-3 32727529 PMC7392835

[B37] Li L. Tian Chye T. Man Karmacharya B. Kumar Govind S. (2012). *Blastocystis* sp.: waterborne zoonotic organism, a possibility? *Parasit. Vectors* 5 1–5. 10.1186/1756-3305-5-130 22741573 PMC3434050

[B38] Li L.-H. Zhou X.-N. Du Z.-W. Wang X.-Z. Wang L.-B. Jiang J.-Y. (2007). Molecular epidemiology of human *Blastocystis* in a village in Yunnan province, China. *Parasitol. Int.* 56 281–286. 10.1016/j.parint.2007.06.001 17627869

[B39] Noradilah S. A. Moktar N. Shahrul Anuar T. Lee I. L. Nor S. Manap A. A. (2017). Molecular epidemiology of Blastocystosis in Malaysia: does seasonal variation play an important role in determining the distribution and risk factors of *Blastocystis* subtype infections in the Aboriginal community? *Parasit. Vectors* 10 360. 10.1186/s13071-017-2294-2 28760145 PMC5537991

[B40] Maloney J. G. da Cunha M. J. R. Molokin A. Cury M. C. Santin M. (2021a). Next-generation sequencing reveals wide genetic diversity of *Blastocystis* subtypes in chickens including potentially zoonotic subtypes. *Parasitol. Res.* 120 2219–2231. 10.1007/s00436-021-07170-3 33904983

[B41] Maloney J. G. Jang Y. Molokin A. George N. J. Santin M. (2021b). Wide genetic diversity of *Blastocystis* in white-tailed deer (*Odocoileus virginianus*) from Maryland, USA. *Microorganisms* 9:1343. 10.3390/microorganisms9061343 34205799 PMC8233720

[B42] Maloney J. G. Lombard J. E. Urie N. J. Shivley C. B. Santin M. (2019). Zoonotic and genetically diverse subtypes of *Blastocystis* in US pre-weaned dairy heifer calves. *Parasitol. Res.* 118 575–582. 10.1007/s00436-018-6149-3 30483890

[B43] Maloney J. G. Molokin A. da Cunha M. J. R. Cury M. C. Dantin M. (2020). *Blastocystis* subtype distribution in domestic and captive wild bird species from Brazil using next generation amplicon sequencing. *Parasite Epidemiol. Control* 9:e00138. 10.1016/j.parepi.2020.e00138 32021915 PMC6995250

[B44] Masuda A. Sumiyoshi T. Ohtaki T. Matsumoto J. (2018). Prevalence and molecular subtyping of *Blastocystis* from dairy cattle in Kanagawa, Japan. *Parasitol. Int.* 67 702–705. 10.1016/J.PARINT.2018.07.005 30009956

[B45] Miller M. A. Pfeiffer W. Schwartz T. (2010). “Creating the CIPRES Science Gateway for Inference of Large Phylogenetic Trees,” in *Proceeding of the SC10 Workshop on Gateway Computing Environments (GCE10)* (Piscataway, NJ: IEEE).

[B46] Mirjalali H. Abbasi M. R. Naderi N. Hasani Z. Mirsamadi E. S. Stensvold C. R. (2017). Distribution and phylogenetic analysis of *Blastocystis* sp. subtypes isolated from IBD patients and healthy individuals in Iran. *Eur. J. Clin. Microbiol. Infect. Dis.* 36 2335–2342. 10.1007/s10096-017-3065-x 28741097

[B47] Osorio-Pulgarin M. I. Higuera A. Beltran-Alzate J. C. Sanchez-Jimenez M. Ramirez J. D. (2021). Epidemiological and molecular characterization of *Blastocystis* infection in children attending daycare centers in Medellin, Colombia. *Biology* 10:669. 10.3390/biology10070669 34356524 PMC8301444

[B48] Padukone S. Selvarathinam A. P. Kumar M. Mandal J. Rajkumari N. Bhat B. V. (2020). Subtype distribution and genetic diversity of *Blastocystis* in asymptomatic and symptomatic individuals from Puducherry, India. *Int. J. Infect. Dis.* 101:431. 10.1016/j.ijid.2020.09.1130

[B49] Parija S. C. Jeremiah S. (2013). *Blastocystis*: taxonomy, biology and virulence. *Trop. Parasitol.* 3 17–25. 10.4103/2229-5070.113894 23961437 PMC3745665

[B50] Parkar U. Traub R. J. Vitali S. Elliot A. Levecke B. Robertson I. (2010). Molecular characterization of Blastocystis isolates from zoo animals and their animal-keepers. *Vet. Parasitol.* 169 8–17. 10.1016/j.vetpar.2009.12.032 20089360

[B51] Pawestri A. R. Thima K. Leetachewa S. Maneekan P. Deesitthivech O. Pinna C. (2021). Seasonal prevalence, risk factors, and one health intervention for prevention of intestinal parasitic infection in underprivileged communities on the Thai-Myanmar border. *Int. J. Infect. Dis.* 105 152–160. 10.1016/J.IJID.2021.02.015 33581366

[B52] Peña S. Carrasco G. Rojas P. Castillo D. Ozaki L. S. Mercado R. (2020). Determination of subtypes of *Blastocystis* sp. in chilean patients with and without inflammatory bowel syndrome, A preliminary report. *Parasite Epidemiol. Control* 8:e00125. 10.1016/J.PAREPI.2019.E00125 31890923 PMC6926359

[B53] Poirier P. Wawrzyniak I. Albert A. El Alaoui H. Delbac F. Livrelli V. (2011). Development and evaluation of a real-time PCR assay for detection and quantification of *Blastocystis* parasites in human stool samples: prospective study of patients with hematological malignancies. *J. Clin. Microbiol.* 49 975–983. 10.1128/JCM.01392-10 21177897 PMC3067686

[B54] Poirier P. Wawrzyniak I. Vivares C. P. Delbac F. El Alaoui H. (2012). New insights into *Blastocystis* spp.: a potential link with irritable bowel syndrome. *PLoS Pathogens* 8:31002545. 10.1371/journal.ppat.1002545 22438803 PMC3305450

[B55] Ramírez J. D. Sánchez L. V. Bautista D. C. Corredor A. F. Flórez A. C. Stensvold C. R. (2014). *Blastocystis* subtypes detected in humans and animals from Colombia. *Infect. Genet. Evol.* 22 223–228. 10.1016/J.MEEGID.2013.07.020 23886615

[B56] Riabi R. T. Mirjalali H. Haghighi A. Rostami Nejad M. Pourhoseingholi M. A. Poirier P. (2018). Genetic diversity analysis of *Blastocystis* subtypes from both symptomatic and asymptomatic subjects using a barcoding region from the 18S rRNA gene. *Infect. Genet. Evol.* 61 119–126. 10.1016/J.MEEGID.2018.03.026 29608961

[B57] Richard R. L. Ithoi I. Azlan M. Majid A. Yusoff W. Sulaiman W. (2016). Monitoring of waterborne parasites in two drinking water treatment plants: a study in Sarawak, Malaysia. *Int. J. Environ. Res. Public Health* 13:641. 10.3390/ijerph13070641 27367710 PMC4962182

[B58] Roberts T. Stark D. Harkness J. Ellis J. (2013). Subtype distribution of *Blastocystis* isolates from a variety of animals from New South Wales, Australia. *Vet. Parasitol.* 196 85–89. 10.1016/J.VETPAR.2013.01.011 23398989

[B59] Scanlan P. D. Knight R. Song S. J. Ackermann G. Cotter P. D. (2016). Prevalence and genetic diversity of *Blastocystis* in family units living in the United States. *Infect. Genet. Evol.* 45 95–97. 10.1016/J.MEEGID.2016.08.018 27545648

[B60] Scanlan P. D. Stensvold C. R. Rajilic-Stojanovi C. M. Heilig H. G. H. J. De Vos W. M. O’toole P. W. (2014). The microbial eukaryote *Blastocystis* is a prevalent and diverse member of the healthy human gut microbiota. *FEMS Microbiol. Ecol.* 90 326–330. 10.1111/1574-6941.12396 25077936

[B61] Scicluna S. M. Tawari B. Clark C. G. (2006). DNA barcoding of *Blastocystis*. *Protist* 157 77–85. 10.1016/J.PROTIS.2005.12.001 16431158

[B62] Shirvani G. Fasihi-Harandi M. Raiesi O. Bazargan N. Zahedi M. J. Sharifi I. (2020). Prevalence and molecular subtyping of *Blastocystis* from patients with irritable bowel syndrome, inflammatory bowel disease and chronic urticaria in Iran. *Acta Parasitol.* 65 90–96. 10.2478/s11686-019-00131-y 31602552

[B63] Stamatakis A. (2006). RAxML-VI-HPC: maximum likelihood-based phylogenetic analyses with thousands of taxa and mixed models. *Bioinformatics (Oxford, England)* 22 2688–2690. 10.1093/bioinformatics/btl446 16928733

[B64] Stensvold C. R. Alfellani M. Clark C. G. (2012). Levels of genetic diversity vary dramatically between *Blastocystis* subtypes. *Infect. Genet. Evol.* 12 263–273. 10.1016/J.MEEGID.2011.11.002 22116021

[B65] Stensvold C. R. Alfellani M. A. Nørskov-Lauritsen S. Prip K. Victory E. L. Maddox C. (2009a). Subtype distribution of *Blastocystis* isolates from synanthropic and zoo animals and identification of a new subtype. *Int. J. Parasitol.* 39 473–479. 10.1016/J.IJPARA.2008.07.006 18755193

[B66] Stensvold C. R. Nielsen H. V. Mølbak K. Smith H. V. (2009b). Pursuing the clinical significance of *Blastocystis* diagnostic limitations. *Trends Parasitol.* 25 23–29. 10.1016/j.pt.2008.09.010 19013108

[B67] Stensvold C. R. Clark C. G. (2016). Current status of *Blastocystis*: a personal view. *Parasitol. Int.* 65 763–771. 10.1016/J.PARINT.2016.05.015 27247124

[B68] Stensvold C. R. Clark C. G. (2020). Pre-empting Pandora’s box: *blastocystis* subtypes revisited. *Trends Parasitol.* 36 229–232. 10.1016/j.pt.2019.12.009 32001133

[B69] Stensvold C. R. Nielsen H. V. (2012). Comparison of microscopy and PCR for detection of intestinal parasites in Danish patients supports an incentive for molecular screening platforms. *J. Clin. Microbiol.* 50 540–541. 10.1128/JCM.06012-11 22090410 PMC3264173

[B70] Stensvold C. R. Tan K. S. W. Clark C. G. (2020). Blastocystis. *Trends Parasitol.* 36 315–316. 10.1016/j.pt.2019.12.008 32001134

[B71] Stensvold C. R. van der Giezen M. (2018). Associations between gut microbiota and common luminal intestinal parasites. *Trends Parasitol.* 34 369–377. 10.1016/J.PT.2018.02.004 29567298

[B72] Suresh K. Smith H. V. Tan T. C. (2005). Viable *Blastocystis* cysts in Scottish and Malaysian sewage samples. *Appl. Environ. Microbiol.* 71 5619–5620. 10.1128/AEM.71.9.5619-5620.2005 16151162 PMC1214661

[B73] Tan K. S. W. (2008). New insights on classification, identification, and clinical relevance of *Blastocystis* spp. *Clin. Microbiol. Rev.* 21 639–665. 10.1128/CMR.00022-08 18854485 PMC2570156

[B74] Tan K. S. W. Mirza H. Teo J. D. W. Wu B. Macary P. A. (2010). Current views on the clinical relevance of *Blastocystis* spp. *Curr. Infect. Dis. Rep.* 12 28–35. 10.1007/s11908-009-0073-8 21308496

[B75] Tito R. Y. Chaffron S. Caenepeel C. Lima-Mendez G. Wang J. Vieira-Silva S. (2019). Gut microbiota population-level analysis of *Blastocystis* subtype prevalence and variation in the human gut microbiota. *Gut* 68 1180–1189. 10.1136/gutjnl-2018-316106 30171064 PMC6582744

[B76] Tsaousis A. D. Hamblin K. A. Elliott C. R. Young L. Rosell-Hidalgo A. Gourlay C. W. (2018). The human gut colonizer *Blastocystis* respires using complex II and alternative oxidase to buffer transient oxygen fluctuations in the gut. *Front. Cell. Infect. Microbiol.* 8:371. 10.3389/fcimb.2018.00371 30406045 PMC6204527

[B77] Tsaousis A. D. Ollagnier De Choudens S. Gentekaki E. Long S. Gaston D. Stechmann A. (2012). Evolution of Fe/S cluster biogenesis in the anaerobic parasite *Blastocystis*. *Proc. Natl. Acad. Sci. U.S.A.* 109 10426–10431. 10.1073/pnas.1116067109 22699510 PMC3387072

[B78] Udonsom R. Prasertbun R. Mahittikorn A. Mori H. Changbunjong T. Komalamisra C. (2018). *Blastocystis* infection and subtype distribution in humans, cattle, goats, and pigs in central and western Thailand. *Infect. Genet. Evol.* 65 107–111. 10.1016/j.meegid.2018.07.007 30003970

[B79] Wang J. Gong B. Yang F. Zhang W. Zheng Y. Liu A. (2018). Subtype distribution and genetic characterizations of *Blastocystis* in pigs, cattle, sheep and goats in northeastern China’s Heilongjiang Province. *Infect. Genet. Evol.* 57 171–176. 10.1016/J.MEEGID.2017.11.026 29196130

[B80] Wang W. Owen H. Traub R. J. Cuttell L. Inpankaew T. Bielefeldt-Ohmann H. (2014). Molecular epidemiology of *Blastocystis* in pigs and their in-contact humans in Southeast Queensland, Australia, and Cambodia. *Vet. Parasitol.* 203 264–269. 10.1016/j.vetpar.2014.04.006 24785292

[B81] Waters E. Ahmed W. Hamilton K. A. Plaksins D. Stark D. (2019). Protozoan pathogens *Blastocystis* and *Giardia* spp. in roof-harvested rainwater: the need to investigate the role of the common brushtail possum (*Trichosurus vulpecula*) and other potential sources of zoonotic transmission. *J. Water, Sanit. Hyg. Dev.* 9 780–785. 10.2166/washdev.2019.064

[B82] Wongthamarin K. Siricharoenthai P. Ountaveesap M. Tangpanitansook S. Mungthin M. Piyaraj P. (2018). Drinking unboiled water is the risk factor of *Blastocystis* incidence in rural community Thailand from prospective cohort study. *Rev. Epidemiol. Sante Publique* 66:S391. 10.1016/J.RESPE.2018.05.422

[B83] Yan Y. Su S. Ye J. Lai X. Lai R. Liao H. (2007). *Blastocystis* sp. subtype 5: a possibly zoonotic genotype. *Parasitol. Res.* 101 1527–1532. 10.1007/s00436-007-0672-y 17665214

[B84] Yoshikawa H. Abe N. Wu Z. (2004). PCR-based identification of zoonotic isolates of *Blastocystis* from mammals and birds. *Microbiology* 150 1147–1151. 10.1099/mic.0.26899-0 15133074

[B85] Yoshikawa H. Koyama Y. Tsuchiya E. Takami K. (2016). *Blastocystis* phylogeny among various isolates from humans to insects. *Parasitol. Int*. 65 750–759. 10.1016/j.parint.2016.04.004 27091546

[B86] Yoshikawa H. Wu Z. Howe J. Hashimoto T. Geok-Choo N. Tan K. S. W. (2007). Ultrastructural and phylogenetic studies on *Blastocystis* isolates from cockroaches. *J. Eukaryot. Microbiol.* 54 33–37. 10.1111/j.1550-7408.2006.00141 17300516

[B87] Yowang A. Tsaousis A. D. Chumphonsuk T. Thongsin N. Kullawong N. Popluechai S. (2018). High diversity of *Blastocystis* subtypes isolated from asymptomatic adults living in Chiang Rai. *Thailand. Infect. Genet. Evol.* 65 270–275. 10.1016/j.meegid.2018.08.010 30118872

[B88] Zhao G. H. Hu X. F. Liu T. L. Hu R. S. Yu Z. Q. Yang W. B. (2017). Molecular characterization of *Blastocystis* sp. in captive wild animals in Qinling Mountains. *Parasitol. Res.* 116 2327–2333. 10.1007/s00436-017-5506-y 28540508

[B89] Zhu W. Tao W. Gong B. Yang H. Li Y. Song M. (2017). First report of *Blastocystis* infections in cattle in China. *Vet. Parasitol.* 246 38–42. 10.1016/J.VETPAR.2017.09.001 28969778

