# Supplementary material for: Blastocystis One Health Approach in a Rural Community of Northern Thailand: Prevalence, Subtypes and Novel Transmission Routes
Source: Front Microbiol. 2021 Dec 9;12:746340. doi: 10.3389/fmicb.2021.746340 (PMC8696170; doi:10.3389/fmicb.2021.746340)

Blastocystis One Health approach in a rural community of northern Thailand: prevalence, subtypes and novel transmission routes

Vasana Jinatham[1], Sadiya Maxamhud[2], Siam Popluechai[1,3], Anastasios D. Tsaousis[2]* and Eleni Gentekaki[1,3]*

Supplementary Material

Supplementary Figure S1: Typical rain collection vessel used to collect rain water in the studied community.

Supplementary Figure S2: Water-dispensing machine, which supplies water to the community

Supplementary Figure S3: A. Water treatment facility of the community studied. **B.** River, which supplies most of the water used by the community. **C.** water towers with taps at the bottom to facilitate water access

Supplementary Figure S4: A. River stream overgrown with morning glory. **B.** Artificial pond lined with soil used for fish farming. **C.** Cement contained used to temporarily hold excess fish and amphibians

Supplementary Figure S5: Soil sampling sites used in this study.

Supplementary Figure S6: Riverbank sampling sites.

Supplementary Figure S1

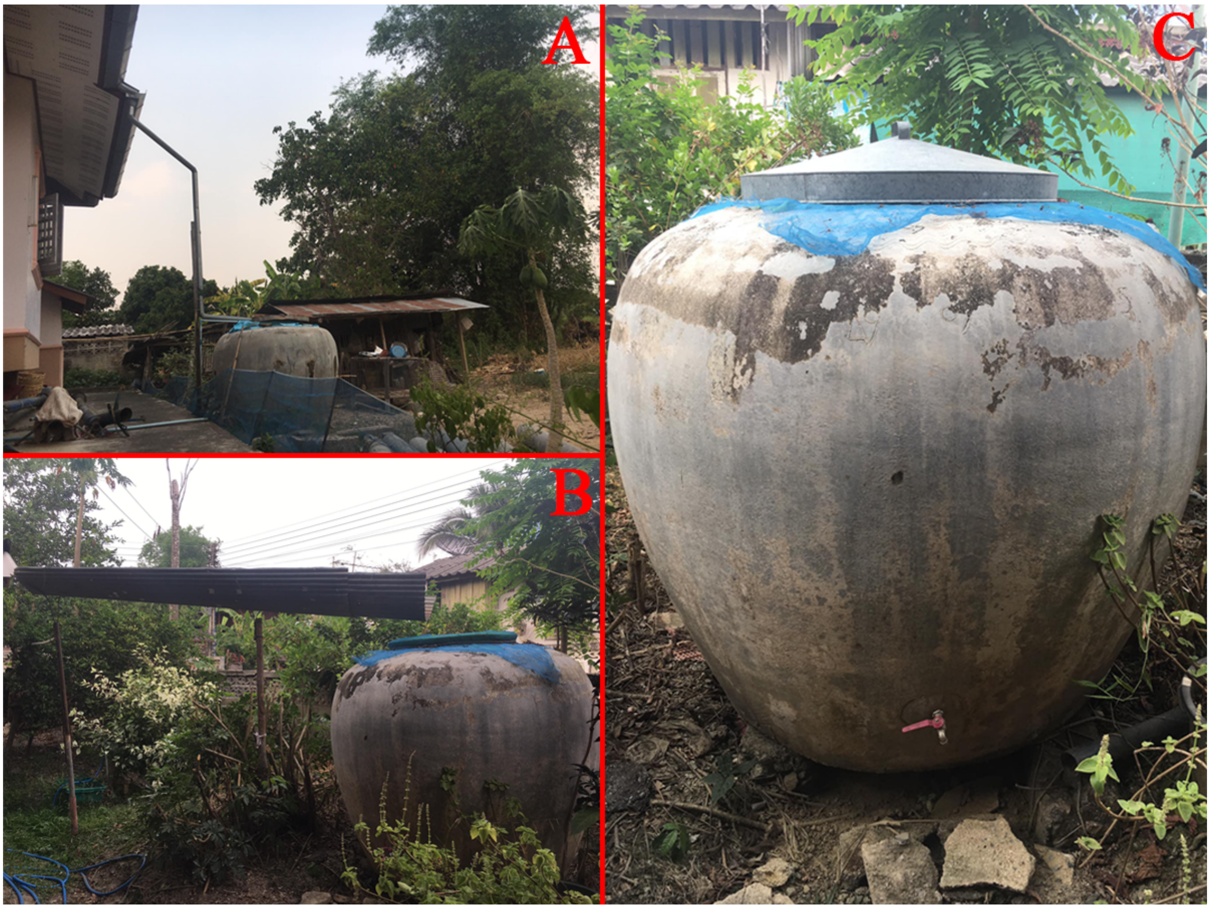

Supplementary Figure S2

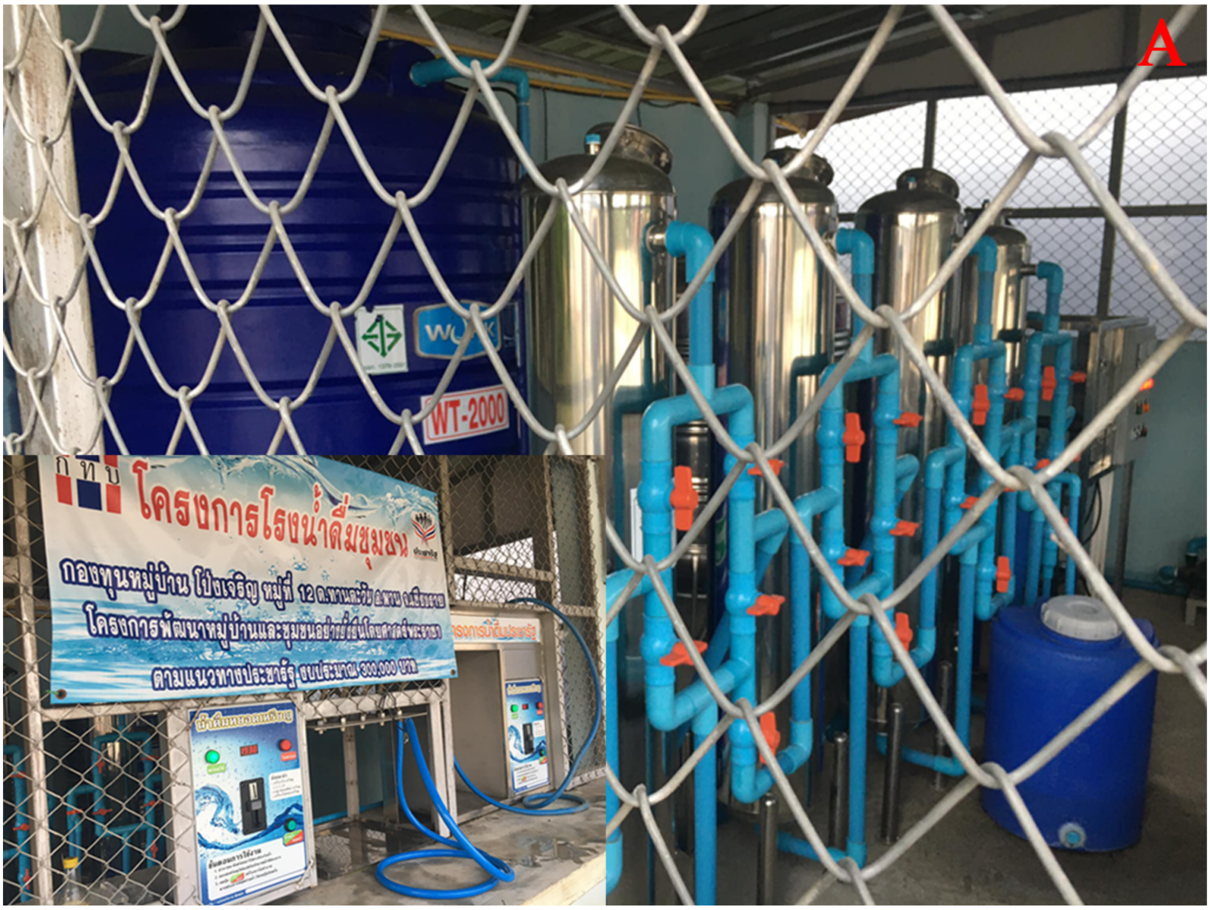

Supplementary Figure S3

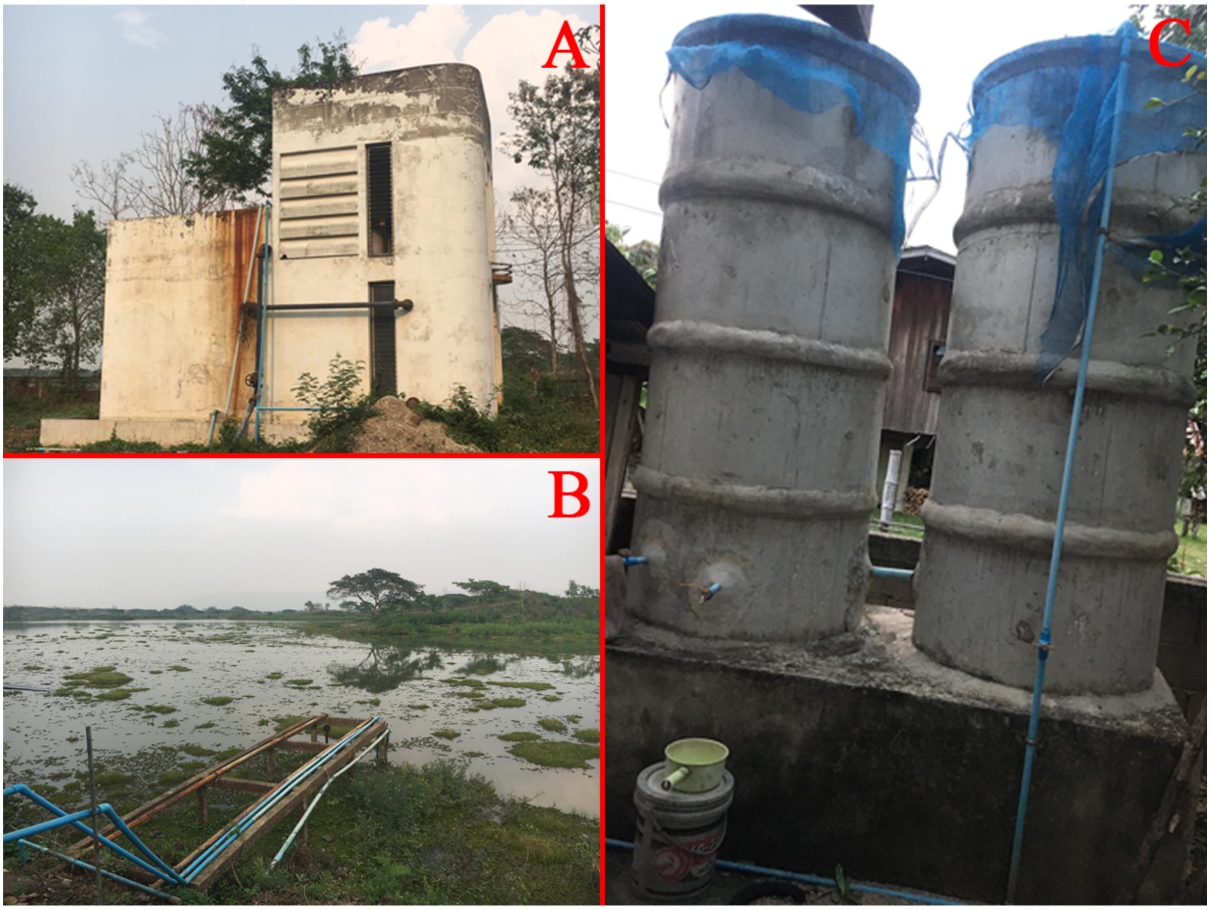

Supplementary Figure S4

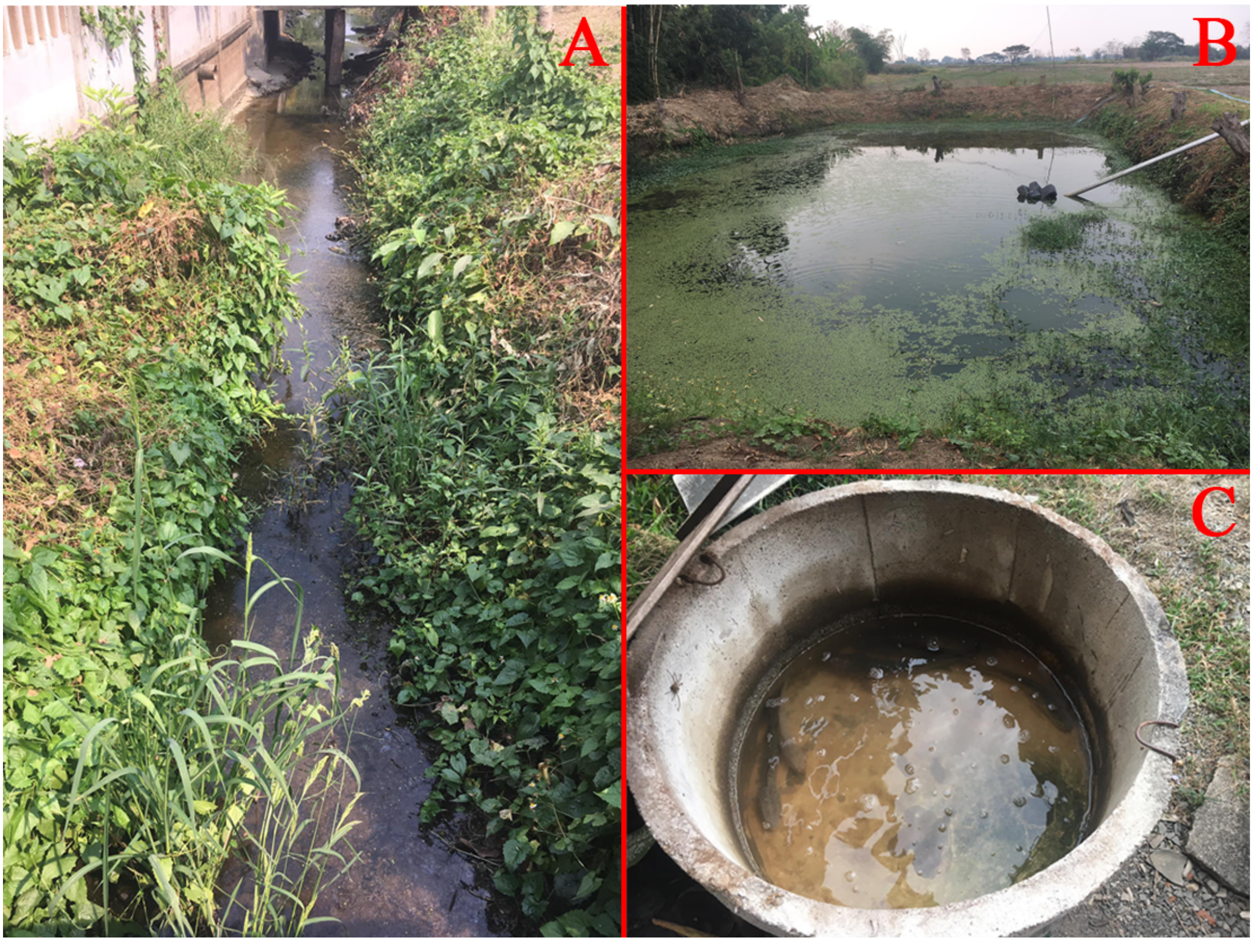

Supplementary Figure S5

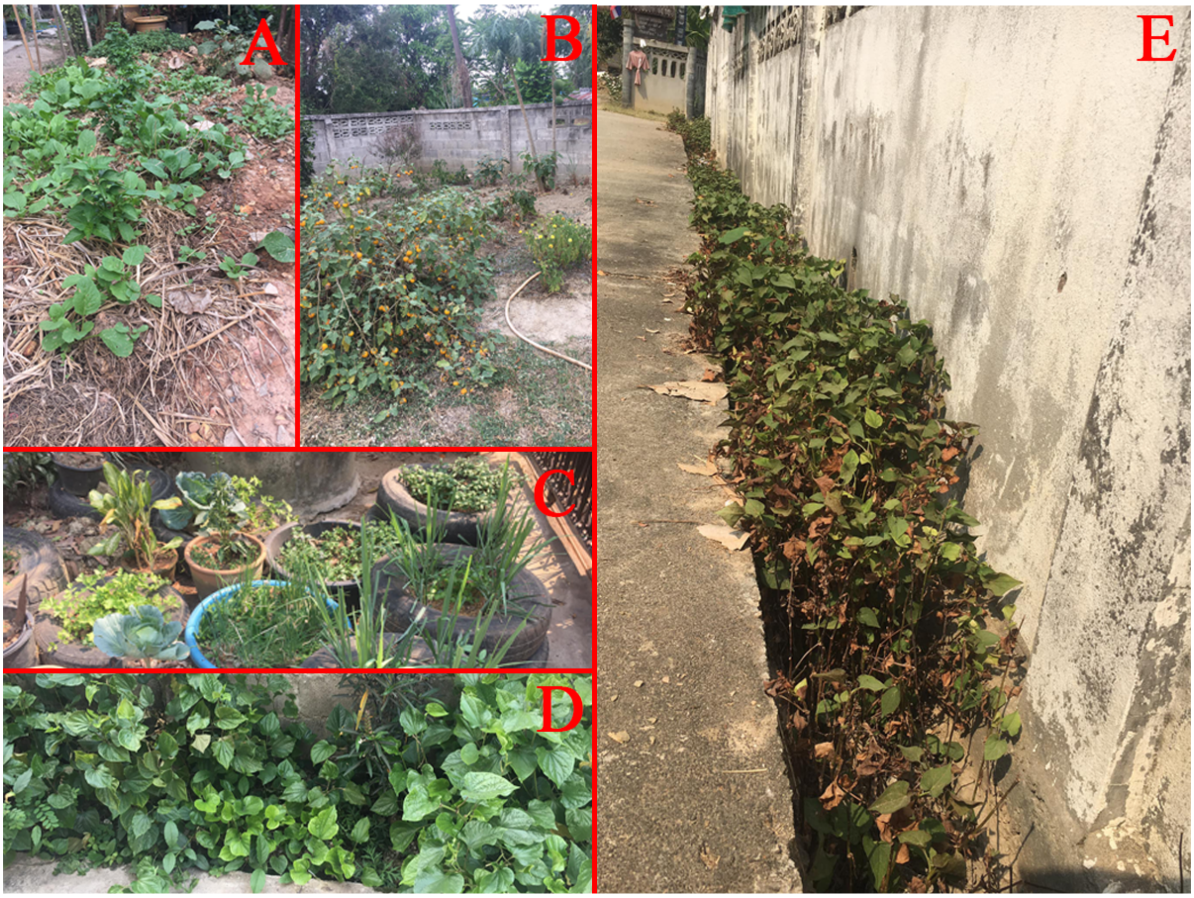

Supplementary Figure S6

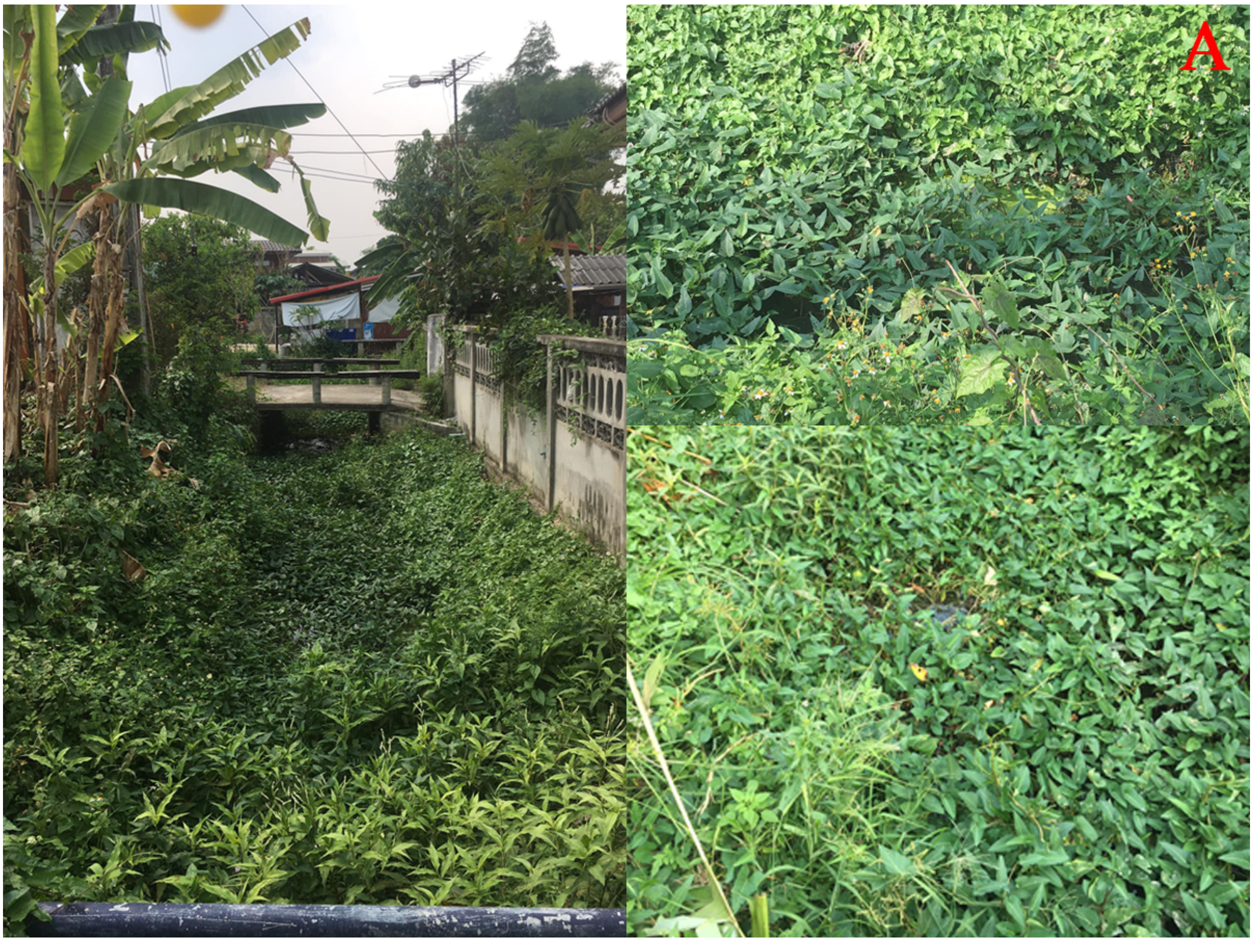

Supplement: Supplementary Material 1 — Includes Supplementary Figures 1–6, which are images from the collection sites. [file Data_Sheet_1.pdf]
